# Integrating virtual patient-based learning into temporomandibular disorder education improves educational and behavioral outcomes

**DOI:** 10.1186/s12909-026-09388-0

**Published:** 2026-05-09

**Authors:** Linda Sangalli, Elizabeth Manning, Aidai Abdykarimova, Olivia Gao, Benjamin Lok, James Hawkins, Craig S. Miller

**Affiliations:** 1 College of Dental Medicine – Illinois, Midwestern University, Downers Grove, Illinois USA; 2 Harper College, Palatine, Illinois USA; 3 Computer & Information Science & Engineering, University of Florida, Gainesville, Florida USA; 4 Naval Postgraduate Dental School, Naval Medical Leader and Professional Development Command, Uniformed Services University of the Health Sciences Postgraduate Dental College, Bethesda, Maryland USA; 5 College of Dentistry, University of Kentucky, Lexington, Kentucky USA

**Keywords:** Predoctoral dental education, Behavioral changes, ORBIT model, Temporomandibular disorders, Digital learning

## Abstract

**Background:**

Predoctoral education in temporomandibular disorders (TMD) often provides limited opportunities for clinical exposure, which may hinder students’ competence and confidence. This study aimed to assess the educational and behavioral impact of a TMD-specific virtual patient-based learning (VPBL) intervention on dental students’ knowledge, behavioral intention for future practice, and confidence levels when integrated into a predoctoral TMD curriculum.

**Methods:**

Seventy-seven third-year dental students at a large US predoctoral program participated in this prospective pre-post study. Initially, students received a 3-hour didactic TMD lecture by an orofacial pain specialist. One week later, they engaged in a 2-h faculty-moderated VPBL session incorporating educational videos and virtual patient cases based on Diagnostic Criteria for TMD. Participants completed assessments before and after the VPBL session, measuring TMD-related knowledge (10-item clinical-based questions), behavioral intention to incorporate TMD screening and management into practice (2 items), and confidence (validated 11-item questionnaire). Pre/post changes were analyzed using paired t-tests, Wilcoxon signed-rank, and McNemar tests.

**Results:**

Mean knowledge scores improved from 4.1 ± 1.9 to 5.9 ± 1.9 (*p*<0.001), with 41.6% increasing to 75.3% of participants achieving a 50% knowledge threshold (*p*<0.001). Confidence increased from 3.6 ± 1.9 to 6.2 ± 1.6 (*p*<0.001), with the proportion of participants reaching 50% sufficient confidence growing from 20.8% to 72.7% (*p*<0.001). Improvements were also observed in behavioral intention to integrate TMD screening and management into future practice (*p*<0.001).

**Conclusions:**

Integrating a TMD-specific VPBL intervention with didactic teaching was associated with short-term improvements in students’ knowledge, confidence, and positive shifts in behavioral intention, supporting VPBL plausibility as an adjunct to predoctoral TMD education.

**Trial registration:**

NCT07203456 (registration date: 09/24/2025).

**Supplementary Information:**

The online version contains supplementary material available at 10.1186/s12909-026-09388-0.

## Introduction

Preparing students across the health professions to manage complex clinical conditions remains a persistent challenge in undergraduate education, particularly in domains characterized by limited clinical exposure and diagnostic complexity. Temporomandibular disorders (TMD), a group of heterogeneous conditions involving pain and/or dysfunction of the temporomandibular joint (TMJ), masticatory muscles, and/or associated structures [1], exemplify such a domain. TMD poses diagnostic and management challenges across multiple health professions, including medicine, physical therapy, behavioral health, and dentistry, with dentists often serving as the first-line providers responsible for initial screening, diagnosis, and management. Despite this expectation, accumulating evidence suggests that many dental graduates feel underprepared and lack confidence in managing TMD-related care [2–10]. This gap in preparedness has profound implications for patient outcomes, referral patterns, and healthcare utilization [11, 12].

A central contributor to this educational gap is the structure of predoctoral TMD training within dental curricula. TMD education is often delivered predominantly through didactic instruction, with limited opportunities for direct patient exposure [13, 14]. While this approach may resemble training in other dental disciplines [15], it is particularly problematic for TMD, as patients with TMD most commonly present to general dental practitioners rather than to specialists for initial care [12].This pattern likely reflects multiple factors, including the relatively recent recognition of orofacial pain as a dental specialty by the American Dental Association in 2020 [16], the limited workforce of orofacial pain specialists across the nation [17, 18], and the high prevalence of TMD signs and symptoms in the general population, estimated at approximately 30% among adults worldwide [19].

Educational research consistently demonstrated that didactic instruction alone is insufficient to promote clinical preparedness, diagnostic reasoning, and confidence [3, 4, 7, 9, 20, 21], particularly for multifactorial conditions such as TMD. As dental education continues to evolve in response to increasing diagnostic complexity, workforce limitations, and expectations for practice readiness, there is a growing imperative to develop innovative curricular approaches that intentionally enhance diagnostic reasoning, clinical judgment, and confidence, which are competencies that cannot be reliable achieved through didactic instruction alone. Thus, meaningful learning in this context requires repeated exposure to realistic clinical scenarios that allow students to integrate history-taking, examination findings, and diagnostic frameworks [13]. Although the importance of incorporating clinical exposure to TMD patients during training is widely recognized, dental curricula face substantial barriers to providing such experiences. These challenges include limited availability of orofacial pain specialists on faculty, large class sizes that restrict small-group or experiential learning, limited opportunities for students shadowing, and the absence of structured rotations in orofacial pain clinics or postgraduate programs [13, 22]. Collectively, these constraints hinder the ability of predoctoral programs to provide consistent, high-quality clinical exposure to TMD patients.

In response to these educational and structural limitations, innovative instructional approaches that can approximate clinical exposure are urgently needed. Technology-based teaching, such as virtual learning environments, represents a promising strategy to address these challenges by bridging the gap between traditional didactic instruction and real-world clinical encounters [23, 24]. Our research group has developed a Virtual Patient-Based Learning (VPBL) platform featuring the most common TMD and orofacial pain conditions according to the Diagnostic Criteria for TMD (DC/TMD) [25] and the International Classification of Orofacial pain (ICOP) [26]. This VPBL environment offers students a structured and interactive setting to apply foundational knowledge, practice clinical reasoning, and engage in decision-making through simulated patient encounters. By standardizing exposure to common clinical presentations and diagnostic pathways, VPBL serves as an intermediate educational step that may partially compensate for limited access to real TMD patients during training. Prior evaluations of the VPBL platform have demonstrated high levels of acceptability, feasibility, and suitability among students [27] and orofacial pain experts [28]. However, it remains unclear whether digital learning environments specifically designed for TMD translate into meaningful behavioral changes among learners, which are necessary for the adoption of these practices in clinical practice.

The overarching goal of this study was to assess the educational and behavioral impact of this VPBL intervention designed to enhance predoctoral TMD clinical instruction. The primary aim was to evaluate whether exposure to the VPBL educational intervention resulted in meaningful improvements in dental students’ TMD-related knowledge performance. A secondary aim was to assess changes in perceived confidence in TMD-related clinical skills relative to predefined thresholds. We hypothesized that the VPBL educational intervention would result in significant improvements in knowledge performance and confidence. Finally, an exploratory aim was to investigate whether the VPBL intervention influenced students’ behavioral intentions regarding TMD care in future practice. Given the exploratory nature of this aim, a priori hypothesis was not formulated.

## Methods

The methodology and results of this study adhere to the Transparent Reporting of Evaluations with Nonrandomized Designs (TREND) Statement, a guideline recommended for nonrandomized behavioral educational interventions [29].

### Study design

This study consisted of a single-group, pre-post quasi-experimental design. The study was deemed exempt by the Institutional Review Board (IRB) of Midwestern University (IRB-25-0306, 04 June 2025). Informed consent was obtained from all participants in compliance with IRB-approved exempt research procedures.

### Eligibility criteria

Participants were eligible if they were enrolled in the third year of the predoctoral dental program at the College of Dental Medicine-Illinois, Midwestern University, and attended the in-person TMD-specific educational intervention integrated in the TMD curriculum (see Study procedure). Participants were excluded if they were enrolled in a different academic year, did not attend the in-person study-related activity, and/or did not complete all study-related tasks.

### Study procedure

The educational intervention was integrated into the existing TMD curriculum for third-year dental students at the College of Dental Medicine-Illinois, Midwestern University. Core TMD-related didactic content was delivered by an orofacial pain specialist, certified by the American Board of Orofacial Pain (ABOP), through three hours of traditional lecture-based instruction. In according with recommendations from the American Academy of Orofacial Pain (AAOP) Committee on TMD Predoctoral Education [30], the content addressed Domain 2 (Screening, Evaluation, Diagnosis, and Risk Assessment) and Domain 4 (Clinical Decision-making, Treatment Planning, Evidence-Based TMD Management, Communication, and Interdisciplinary Collaboration in Clinical Practice). Students were instructed to review such didactic material in preparation for this educational session. One week later, the same orofacial pain specialist facilitated a two-hour VPBL session delivered in a digital learning environment (see VPBL educational intervention).

Participants completed a pre-intervention assessment prior to the VPBL session to evaluate TMD-related knowledge and behavioral intention for future practice. A post-intervention assessment was administered immediately following completion of the VPBL session to evaluate TMD-related knowledge, behavioral intention for future practice, and change in confidence levels (see Outcome measures).

**Fig. 1:**
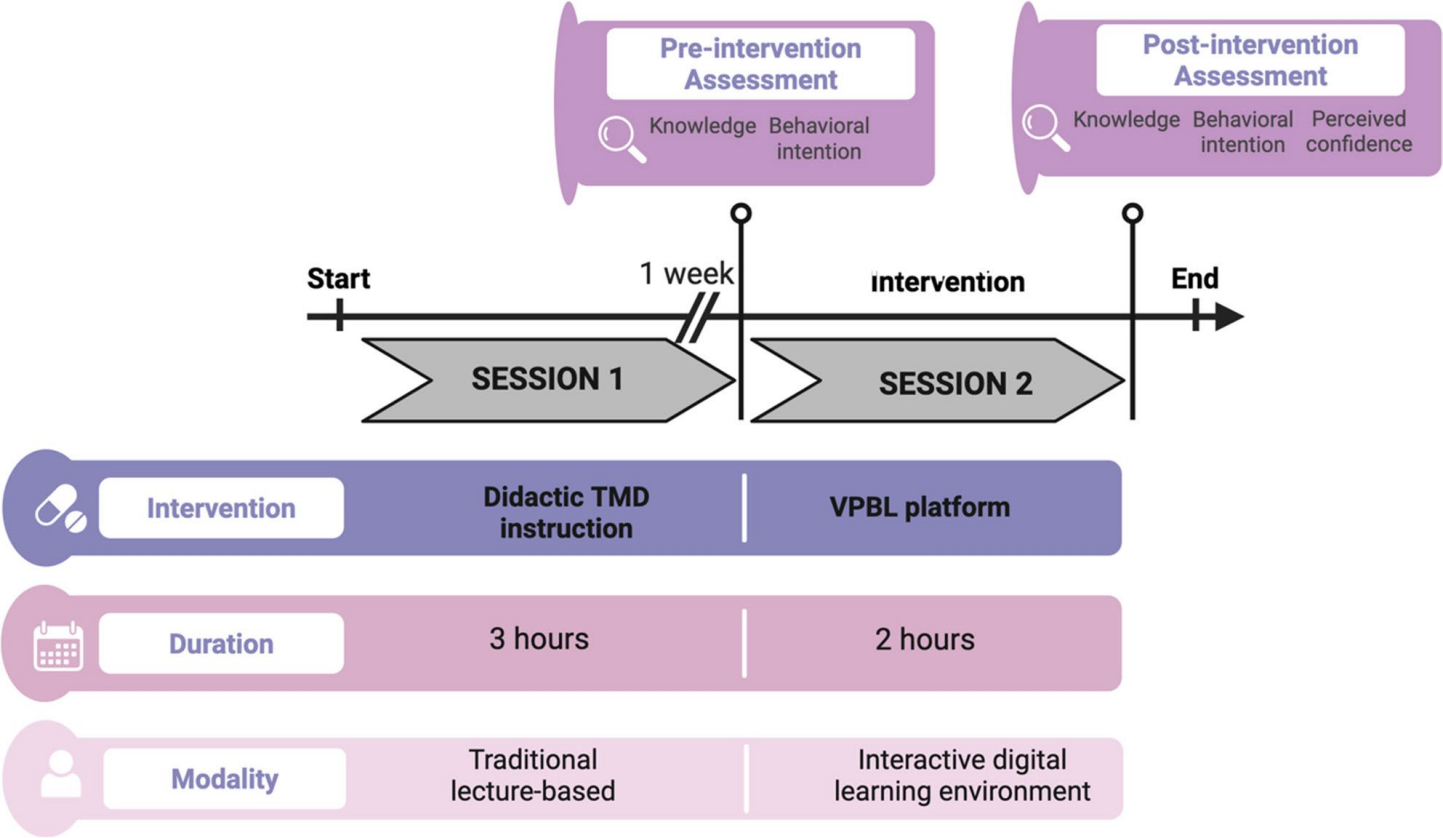
Study procedure

Attendance at the educational intervention was mandatory. Participants received a $40 gift card as compensation for their time. Fig. 1 illustrates the study procedure.

### Virtual patient-based learning (VPBL) educational intervention

As described elsewhere [27, 28], this VPBL consists of four modules (muscular TMD, articular TMD, mixed TMD, and non-TMD) comprising a total of 20 virtual patients (i.e., 5 per module), presented in order of increasing clinical complexity. Each virtual patient guides learners through a structured orofacial pain interview, including history taking, biopsychosocial assessment, medical history, clinical examination, and radiographic findings. After reviewing each case, the users are prompted to select the most appropriate diagnosis and management strategies through multiple-choice quizzes with immediate corrective feedback. Compared with earlier VPBL prototypes [27, 28], the version tested in this study was refined to incorporate educational videos demonstrating TMD clinical examination procedures, diagnostic/trigger point injections, and comprehensive history-taking interviews.

During the two-hour educational intervention, participants engaged with 8 virtual patient case scenarios (i.e., two from each module).

### Outcome measures

The assessment tool is provided in Supplemental File 1.

#### Primary outcome: TMD-related knowledge performance

TMD-related knowledge was assessed at pre- and post-intervention using a 10-item multiple-choice questionnaire designed to reflect the content and structure of the course final examination. Items were developed by subject matter experts in orofacial pain and experts in dental education, and aligned with core domains, including TMD diagnoses, clinical examination procedures, pain descriptors obtained during history taking, evidence-based treatments, indication of trigger point injections, diagnostic definitions based on the Diagnostic Classification for TMD (DC/TMD), and interpretation of radiographic findings. Content validity was established through expert review to ensure alignment with curricular objectives and standards for predoctoral dental education set by the Commission on Dental Accreditation (CODA) [31]. Each item was scored as 1 (correct) or 0 (incorrect), yielding a total score ranging from 0 to 10, with higher scores indicating greater knowledge. Internal consistency reliability of the scale was assessed using Cronbach’s alpha (α = 0.57). At post-intervention assessment, items and answer choices were presented in randomized order to reduce recall bias.

#### Secondary outcome: confidence level in TMD-related skills 

At post-intervention, confidence level in TMD-related skills was measured using a previously validated 11-item questionnaire [3]. A retrospective pre-post approach was employed to reduce response-shift bias and over- or underestimation of baseline confidence. Items assessed confidence in TMD screening, diagnosis and management, patient education regarding etiological factors and self-care strategies, treatment need, occlusal appliances adjustment, and performance of diagnostic injections, and interprofessional referral and communication.

#### Exploratory outcome: behavioral intention for future practice

Behavioral intention to screen and manage patients with TMD was assessed at both pre- and post-intervention using two items informed by prior literature [32] and selected to reflect clinically relevant decision-making and intent of future action. Responses were recorded on a 6-point Likert scale ranging from 1 (“Very likely”) to 5 (“Surely unlikely”), with an additional option of 6 (“Unsure”).

Finally, participants reported the number of TMD patients they had previously encountered while shadowing the orofacial pain specialist.

### Sample size

An a priori sample size calculation was not performed, as the study aimed to include all eligible third-year dental students enrolled at the College of Dental Medicine at Midwestern University. Based on the primary outcome of change in knowledge performance scores between pre- and post-intervention, a post-hoc power analysis was conducted for a paired t-test using the observed mean difference of 1.8 points on a 0–10 scale and a standard deviation (SD) of 1.9. With 77 participants providing complete responses and a two-tailed significance level of 0.05, the study had > 99% power to detect a significant change in performance scores.

### Statistical analysis

Prior to analysis, the database was examined for completeness. Missing values were handled using casewise (listwise) deletion.

Normality distribution was assessed with Shapiro-Wilk test and visual inspection of distributions (histograms and Q-Q plots) prior to inferential testing. Accordingly, pre-post changes in perceived confidence were evaluated using paired t-tests (as found to be normally distributed) and changes in TMD-related knowledge performance were assessed using Wilcoxon singed-rank test (as found not normally distributed with Shapiro-Wilk *p* < 0.05). Effect sizes were computed using Cohen’s *d* for parametric tests and *r* for nonparametric tests and interpreted according to conventional thresholds. Consistent with the ORBIT model’s emphasis on predefined, clinically meaningful benchmarks in early-phase studies [33], thresholds for sufficient knowledge and confidence were set a priori at ≥ 70% and ≥ 50%, respectively. The 70% cutoff for knowledge performance reflects conventional benchmarks in educational settings, whereas the 50% confidence threshold was selected based on prior evidence demonstrating a statistically significant increase in TMD screening practices among dental professionals at this level [2]. As prior literature suggested that very few students achieve a 70% threshold in TMD-related knowledge, the analysis was also conducted setting 50% as target for knowledge performance [4]. Given the discrete and bounded nature of the 10-item scale, these thresholds were used for exploratory purposes. Proportions of participants achieving these predefined thresholds for knowledge and confidence were compared between pre- and post- intervention assessments with McNemar’s tests. Pearson’s correlation coefficient was used to test the correlation between knowledge performance score and perceived confidence.

Changes in behavioral intention for future practice between pre- and post-assessment were tested using Bowker’s test of symmetry. Associations between post-intervention confidence and likelihood of screening and/or treating TMD were examined using ordinal logistic regression with a logit link function.

A two-tailed *p* value of < 0.05 was considered statistically significant. Statistical analyses were conducted with SPSS (v31, IBM Corp., Armonk, NY).

## Results

**Fig. 2:**
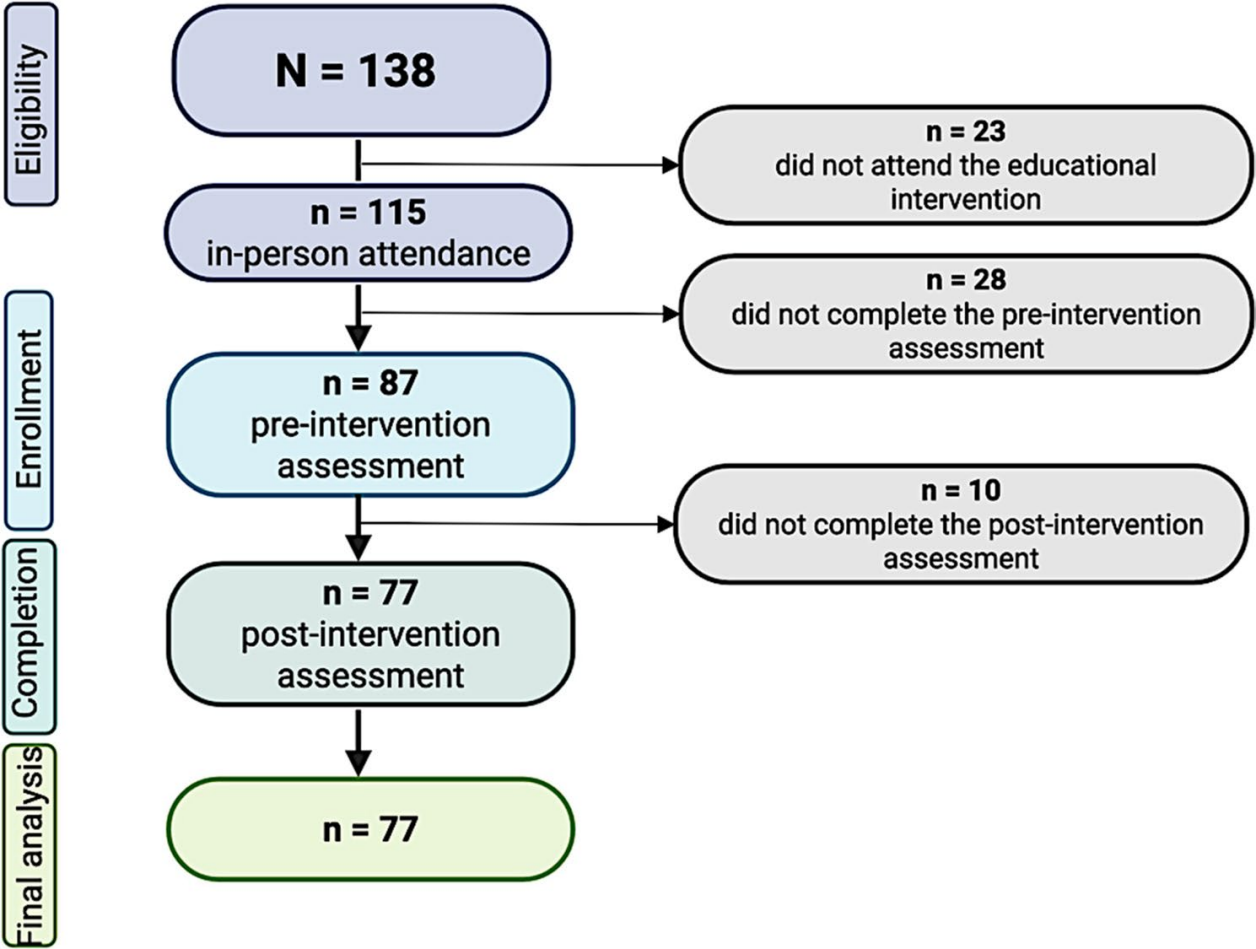
Flowchart of the study participants

Of 138 third-year dental students (64.1% females; 40.7% White, 31.0% Asian, 16.6% Hispanic, 2.8% Black or African American, 8.9% two or more races), 115 attended the educational intervention (83.3%). Of these, 87 (75.7%) returned the pre-intervention assessment and 77 of these 87 (88.5%) completed the post-intervention assessment. Fig. 2 illustrates the participant flowchart. A sensitivity analysis revealed that the participants completing all study-related tasks and those who only completed the pre-intervention assessment did not differ in pre-intervention knowledge and behavioral intention for future practice (Supplemental File 2).

### Change in TMD-related knowledge performance

**Fig. 3:**
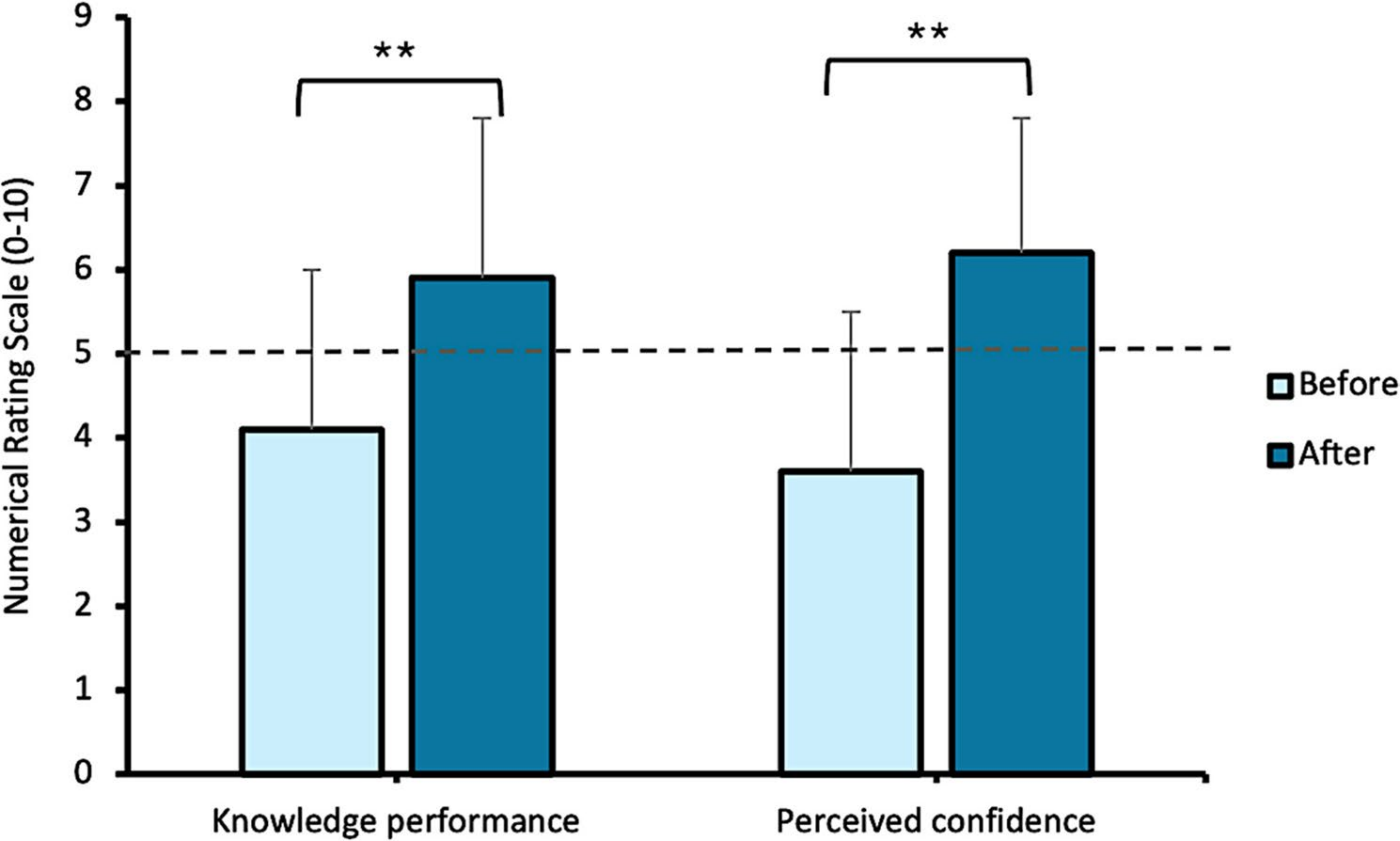
Changes in TMD-related knowledge performance and perceived confidence between pre- and post-assessment. **significant at *p*<.001. The dotted line refers to a 50% sufficient threshold

**Fig. 4:**
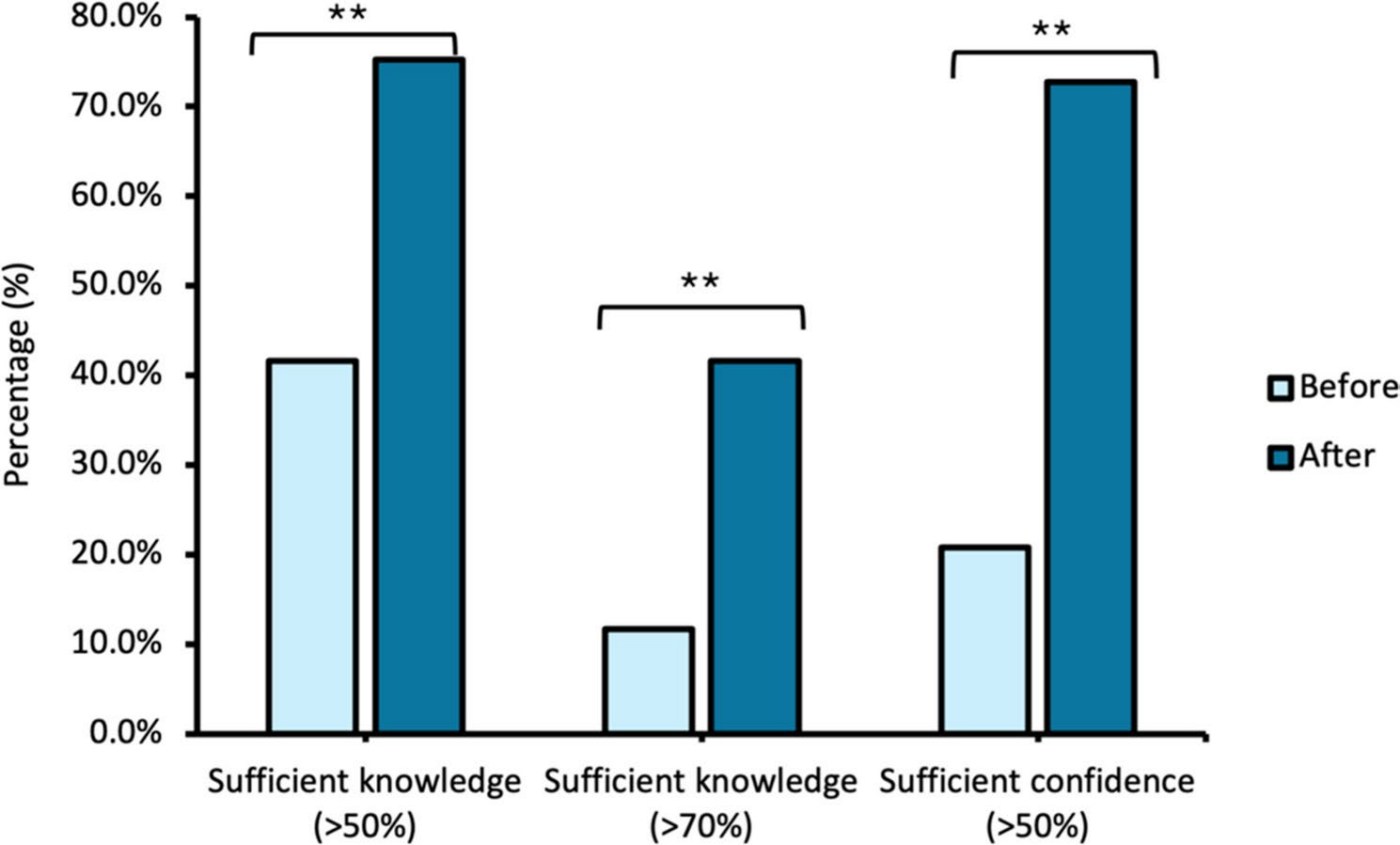
Change in proportion of participants achieving predefined knowledge and confidence thresholds. **significant at *p*<.001

At baseline, participants reported an overall mean knowledge performance of 4.1 ± 1.9, with 41.6% and 11.7% of them achieving a 50% and 70% knowledge threshold, respectively. At post-assessment, the overall mean knowledge performance significantly increased to 5.9 ± 1.9 (*Z*=-6.18, *p* < 0.001, *r* = 0.73, Fig. 3). The proportion of participants achieving a 50% and 70% knowledge threshold significantly increased to 75.3% and 41.6%, respectively (all *p*’s < 0.001, Fig. 4).

### Change in perceived confidence levels in TMD-related skills

Mean confidence levels increased from 3.6 ± 1.9 to 6.2 ± 1.5 (*p* < 0.001, *d* = 1.77, Fig. 3). The proportion of participants achieving a 50% sufficient confidence threshold significantly increased from 20.8% to 72.7% post-assessment (*p* < 0.001, Fig. 4). Perceived confidence positively correlated with knowledge performance scores at both pre- (r(67)=0.264, *p* = 0.031) and post-intervention timepoints (r(67)=0.263, *p* = 0.032).

### Change in behavioral intention for future practice

**Fig. 5:**
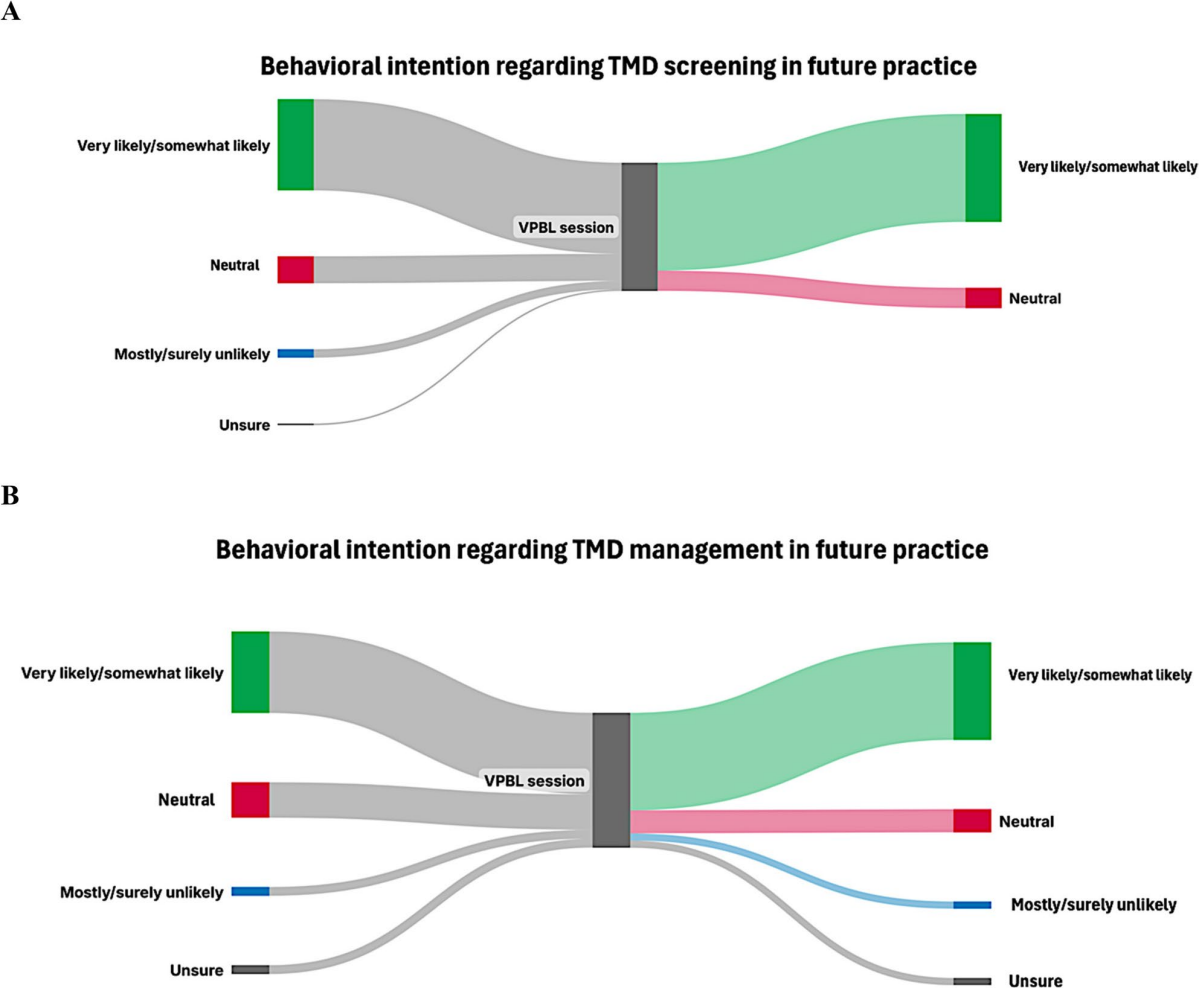
Changes in behavioral intention of TMD screening (**a**) and management (**b**) for future practice between pre- and post-assessment. Sankey diagram: node labels represent the probability associated with each intention category, and the thickness of the connecting flows illustrates how many participants moved between categories from pre- to post-assessment

Exposure to the educational activity was associated with a significant improvement in behavioral intention for future practice. At baseline, 71.1% of participants indicated being very likely/somewhat likely to integrate TMD screening into practice upon graduation, whereas 6.6% reported being mostly/surely unlikely; 21.1% were neutral and 1.3% were unsure. At post-intervention, the proportion of participants reporting that they were very likely/somewhat likely to integrate TMD screening increased to 84.2%, while the remaining 15.8% reporting a neutral intention (*p* < 0.001). More heterogeneity was reported regarding the intention of integrating TMD management into practice upon graduation. At baseline, 60.2% of participants indicated being very likely/somewhat likely; 6.6% reported being mostly/surely unlikely; 26.3% were neutral and 6.6% were unsure. At post-intervention, the proportion of participants reporting being very likely/somewhat likely to integrate TMD management increased to 72.4%, while the remaining 17.1% reported being neutral, 5.3% unsure, and 5.3% mostly/surely unlikely (*p* < 0.001). Fig. 5 illustrates changes in behavioral intention in future practice between pre- and post-assessment. Higher post-intervention confidence was significantly associated with greater likelihood of intending to manage TMD into future practice (B=-0.33, 95% CI -0.63, -0.03, *p* = 0.031), but was not significantly associated with intention to screen for TMD (B=-0.123, 95% CI -0.41, 0.16, *p* = 0.395).

## Discussion

The current study investigated whether a TMD-specific, technology-based teaching based on virtual patients was associated with changes in third-year students’ knowledge, confidence, and behavioral intentions related to TMD care. Our findings suggest that participation in the VPBL intervention was associated with significant and meaningful improvements in knowledge performance and perceived confidence, with evidence of a positive shift in self-reported behavioral intentions regarding TMD care upon graduation.

### Digital patient-based simulations accompanying didactic instructions are associated with improvements in knowledge and perceived confidence in TMD-related skills

In alignment with the ORBIT model, a rigorous, phase-based framework that tests the development of behavioral interventions aimed at improving a chronic healthcare issue, the present investigation consisted of an ORBIT Phase I Refine study (Phase Ib, Mediators) where a within-subject pre-post design evaluates whether the intervention can achieve meaningful predefined thresholds [33]. The milestone of Phase I Refine testing is to achieve predefined clinically meaningful benchmarks rather than proving statistically significant differences compared with standard care. Progression to later stages of testing (Phase II proof-of-concept and pilot studies) is contingent upon meeting predefined thresholds [34]. At baseline, following didactic instruction alone, students demonstrated relatively low knowledge performance, with a mean score of 4.1 on a 0–10 scale, with fewer than 42% of students meeting the minimum threshold of 50%, and only ~ 12% achieving a 70%-knowledge benchmark. These findings may reflect limitations of didactic instruction alone in supporting knowledge acquisition in TMD-related knowledge. Baseline results are slightly lower than similar literature, where 51% of third-year students reached a 50% knowledge cutoff [4], although direct comparisons across studies is limited by differences in assessment tools.

Following the VPBL intervention, knowledge performance increased by nearly 2 points on a 0–10 scale with a large effect size, in alignment with similar research evaluating digital learning environments specifically designed for TMD education [35, 36]. At post-intervention, approximately 75% of students met the 50% knowledge threshold and 42% met the 70% threshold. In this study, both 50% and 70% knowledge thresholds were explored. While a 70% cutoff may better align with commonly used thresholds in health professions education [37], the inclusion of a 50% threshold reflects prior literature demonstrating the difficulty of achieving even minimal competency among predoctoral students in TMD-related education [4]. Despite these improvements, absolute performance remained moderate, with a mean score of 6 on a 0–10 scale. Altogether, these findings suggest that, while the VPBL intervention was associated with meaningful gains, it may be insufficient as a standalone intervention. This interpretation aligns with prior literature that support the importance of repeated exposure, reinforcement, multimodal instructional strategies [4, 38], and clinical application in promoting knowledge construction and retention [3, 4, 32, 39]. Accordingly, future curricular improvements may include greater integration of case-based and problem-based learning, digital environments, small-group discussion, application-based instruction, integrated curricula, and structured clinical reasoning frameworks such as SNAPPS [40, 41]. Collectively, these approaches have been shown to support the development of clinical reasoning, abstract thinking, and critical thinking skills [40]. In addition, participants in this study were third-year students with relatively recent exposure to the materials (i.e., novice learners). As in other areas of dental education, continued progression to later academic year may further support knowledge acquisition and skill development over time [3, 4, 42].

Perceived confidence also increased following the VPBL intervention by 2.6 points on a 0–10 scale (from 3.6 to 6.2 on average), with a large effect size. A 50% confidence threshold was selected as it reflected significantly greater screening practice among dental providers [43]. The proportion of students meeting the 50% confidence threshold increased substantially, from ~ 21% to ~ 73%. Although confidence is subjective and does not equate to clinical competence, confidence in TMD-related skills was positively correlated with knowledge in this study and prior reports [4, 42], and represents a critical determinant of learners’ willingness to translate acquired knowledge into clinical practice after graduation [44].

Beyond improvements in knowledge and confidence, educational interventions should ultimately influence learners’ intention to screen for and manage TMD in future practice. This outcome is particularly relevant given the national shortage of dental providers trained in orofacial pain management [17, 18]. Prior research indicate that students recognize the importance of diagnosing and managing TMD, and intend to incorporate TMD care in their professional careers [45]. In this study, a shift in learners’ self-reported practice intentions was observed following the VPBL intervention, which may indicate alignment between educational exposure and intended clinical behaviors in patient care. However, self-reported intention has been suggested as a weak and imperfect predictor of actual clinical behavior [46]; thus, these findings should be interpreted with caution and confined to reported intentions. Future research should determine whether these changes translate into measurable clinical behaviors in practice.

In accordance with the ORBIT model’s emphasis on progression based on achievement of pre-specified meaningful clinically milestones [33], the current version of the intervention is now positioned to advance to Phase II preliminary testing characterized by randomized clinical trial designs, with hypothesis-driven testing, participant randomization, appropriate control groups receiving standard instruction, and assessment of patient-centered outcomes [33].

### Digital patient-based simulations could help addressing challenges of predoctoral TMD instruction

Predoctoral TMD instruction has made significant progress over the past two decades, evolving from an occlusion-centered perspective to a comprehensive, biopsychosocial approach [13, 14, 47]. Curricula now dedicate substantial more hours to didactic teaching, covering a broader range of content, including less common orofacial pain diagnoses (e.g., neuropathic pain and primary headache) [13]. Emerging research highlights the importance of clinical exposure to TMD patients for knowledge retention and confidence building [3, 4, 48], prompting gradual integration of experiential learning activities such as case-based learning, peer hands-on practice, clinical rotations in orofacial pain clinics or postgraduate programs, and dental patient screening for TMD. Despite these advances, structural challenges remain in predoctoral TMD education. Clinical exposure to TMD patients is highly heterogeneous, reflecting the wide spectrum of orofacial pain presentations [49]. Many dental schools struggle to provide sufficient patient encounters. As a matter of fact, a 2025 national-based study reported that students were exposed to an average of only 1.4 TMD patients during training, with ~ 52% of students lacking any exposure [3]. While even a single patient encounter was found to be sufficient to improve students’ behavioral outcomes [3, 4], this level of exposure is insufficient to master the diverse clinical manifestations of TMD. Moreover, clinical experiences varied widely, ranging from no patient encounters for about 50% of students to 30 patients for less than 1% of students [3]. Limited exposure is often due to a shortage of orofacial pain specialists on faculty and the small number of affiliated postgraduate residency programs (fewer than 15 at the time of the study) [17, 18]. Additionally, the large class sizes typical of dental programs constrain opportunities for small-group or one-on-one learning, which are essential for developing critical reasoning and clinical decision-making skills [40]. In clinically complex conditions like TMD [50], didactic instruction alone cannot replicate the value of real-time patient follow-up: students need to observe whether the management strategies recommended by clinicians are effective in practice. This type of experiential learning requires patient interaction and cannot be taught solely through lectures.

The TMD-specific VPBL platform aims to address these challenges. By incorporating 20 virtual patients, the platform reduces variability in clinical exposure [51], provides standardized scenarios representing common TMD and orofacial pain conditions [27, 52, 53], allows for the gradual progression to more complex cases without overwhelming novice learners, and helps mitigate the lack of trained faculty in many dental schools. While the current version does not yet simulate patient follow-up, digital environments have the potential to incorporate gamification and branching scenarios, allowing students to experience consequences of clinical decisions over time [54]. In this study, the VPBL session was moderated by faculty and delivered to the full third-year class, which limited the self-paced and individualized advantages of digital learning, including critical reasoning challenges embedded in quizzes. Future implementations could deploy the VPBL platform as individual assignments following didactic teaching, to better prepare students for final exams and maximize its educational potential.

Digital learning environments have the potential to mitigate several challenges in TMD education by serving as a complimentary tool to real patient interaction within a safe, controlled setting, much like flight simulators prepare pilots for real-world flying. These environments cannot replace interactions with actual patients, as research demonstrates that authentic patient encounters provide greater authenticity, communicational experiences, emotional expressiveness, and exposure to non-verbal cues that are critical for developing clinical judgement and communication skills [53].

Despite well-established diagnostic criteria for TMD [25], a comprehensive understanding of its multifactorial etiology (e.g., biological, psychosocial, structural and functional aspects of the masticatory system, as well as parafunctional behaviors) [55] that reliably guides individualized treatment selection remains limited. This underscores the importance of framing TMD education not as the transmission of fixed or fully structured knowledge, but as the development of clinical reasoning and critical thinking skills within an evolving evidence base. For predoctoral learners, this includes recognizing the limitations of current knowledge, applying reversible and evidence-based management strategies in straightforward cases, and maintaining a critical and adaptable approach as emerging evidence continue to refine our understanding of TMD mechanisms and treatment.

### Limitations

This study has some limitations. Although the educational intervention was mandatory except for absences justified by medical reasons, participation in the pre- and post-intervention questionnaire was voluntary due to research requirements. As a result, responses were obtained from 76% of attending students. Sensitivity analysis, however, did not show significant differences between students with complete (i.e., both pre- and post-intervention questionnaires) and those with incomplete data (i.e., pre-intervention questionnaire only). Research embedded withing mandatory educational activities may reduce selection bias and improve internal validity compared with studies relying solely on voluntary recruitment [56].

Despite being instructed to prepare for the activity as they would for a final examination, the low pre-intervention knowledge score may reflect inadequate review of the material. Nevertheless, the didactic content was delivered one week prior to the activity, a timeframe generally considered sufficient for knowledge retention. These findings may therefore underscore the challenges students face in acquiring and retaining information through didactic instruction alone. Similarly, the observed improvements in knowledge and confidence following the intervention may partly reflect reinforcement of previously introduced concepts rather than exposure to VPBL alone. Even so, these results emphasize the value of multimodal educational approaches and are consistent with prior literature [4]. The absence of a control group limits the ability to attribute observed changes directly to the VPBL intervention, as test-retest effects [57], Hawthorne effects [58], or reinforcement of prior didactic instruction cannot be excluded [57]. To establish the effectiveness of the VPBL intervention in producing behavioral changes, subsequent Phase II preliminary testing should incorporate randomization and a control group receiving standard instruction (e.g., didactic teaching alone). While this pre-post study evaluated outcomes immediately following the intervention, future research should examine long-term retention of knowledge and sustainability of behavioral changes through longitudinal assessments.

This study also has several strengths. For the first time, a TMD-specific VPBL platform was tested using objective outcomes such as knowledge performance, evaluated using OSCE-style questions. It is also the first study to explore whether exposure to this educational intervention translated into improved intentions to provide better care for TMD patient population. By integrating the VPBL within a mandatory curriculum and employing rigorous assessment tools, this study offers valuable insights into the potential of innovative, technology-based learning to fill the gaps of current predoctoral TMD instruction.

## Conclusions

The integration of a digital patient-based learning into the predoctoral TMD curriculum was associated with significant and meaningful improvements in students’ knowledge and perceived confidence in TMD-related skills, along with positive shifts in their intention to incorporate TMD screening and management into future clinical practice. These findings are encouraging and support progression to more rigorous, hypothesis-driven Phase II testing. 

## Supplementary Information

Supplementary Material 1.

Supplementary Material 2.

## Data Availability

The datasets used and/or analyzed during the current study are available from the corresponding author on reasonable request.
