# Supplementary material for: Integrating virtual patient-based learning into temporomandibular disorder education improves educational and behavioral outcomes
Source: BMC Med Educ. 2026 May 9;26:1063. doi: 10.1186/s12909-026-09388-0 (PMC13330305; doi:10.1186/s12909-026-09388-0)
Supplement: Supplementary file 1 — Supplementary Material 1. [file 12909_2026_9388_MOESM1_ESM.docx]

**Supplemental File 1.** Questionnaire assessment tool.

| **Item** | **Answer options** | | **Assessment timepoint** |
| --- | --- | --- | --- |
| How many TMD patients have you seen so far in your training? | (free text) | | Pre-intervention |
| **Behavioral intention for future practice** | | | |
| 1. How likely are you to integrate TMD screening into your practice upon graduation? | - Very likely - Somewhat likely - Neutral - Mostly unlikely - Surely unlikely - Unsure | | Pre- and post-intervention |
| 2. How likely are you to integrate TMD treatment into your practice upon graduation? | - Very likely - Somewhat likely - Neutral - Mostly unlikely - Surely unlikely - Unsure | |  |
| **TMD-related knowledge performance** | | | |
| 1. Which muscular condition is defined as “pain upon palpation of a muscle that spreads beyond the palpation site but remains within the muscle boundary”? | - Local myalgia - Myofascial pain with spreading - Myofascial pain with referral - TMJ | | Pre- and post-intervention |
| 2. During the palpation of the masseter muscle, you provoke the patient’s familiar pain. How would you proceed to test if there is referred pain from the masseter elsewhere? | - By palpating the masseter muscle in an intermittent fashion - By removing the pressure on the masseter muscle and asking the patient if they feel the pain somewhere else - By informing the patient that you are going to maintain the pressure on the muscle for 5 seconds to see if the pain is felt elsewhere - By applying much more pressure to the masseter muscle | |  |
| 3. Which of the following descriptors are most likely to describe TMD-related pain? | - Pulsating and throbbing - Pins and needles - Ache and dull - Electric | |  |
| 4. In which of the following diagnoses is a trigger point injection indicated? (select all that apply) | - Arthralgia of the left TMJ - Myofascial pain of the right temporalis radiating within the boundary of the muscle - Local myalgia of the right masseter - Myofascial pain of the left masseter referring to the ear | |  |
| 5. You tentatively diagnose your patient with myofascial pain of the left masseter muscle, radiating to the upper left molars. To confirm your diagnosis, you propose to do a trigger point injections in the left masseter. What kind of anesthesia would you use? | - Lidocaine 1% with epinephrine 1:100,000 - Mepivacaine 3% without epinephrine - Bupivacaine 0.25% for its long-lasting effect - Saline 0.9% for its wet needling effect | |  |
| 6. From your history taking, you learn that your patient has dull pain on bilateral pre-auricular area (6/10 of intensity), triggered by opening and chewing gum. On clinical exam, you reproduce the patient pain by palpating on masseters (which radiates to the ears). The patient has also a clicking sound on the left TMJ with opening. What is the most appropriate muscular diagnosis? | - Myofascial pain with referral - Local myalgia - Disc displacement with reduction - Myofascial pain with spreading | |  |
| 7. Which of the following are appropriate management options for a 38-year-old female patient diagnosed with arthralgia of the right TMJ? | - Trigger point injections, short cycle of NSAIDS - Botulinum toxin injections, short cycle of NSAIDS - Stabilization appliance, physical therapy, short cycle of NSAIDS - Referral to OMFS for total joint replacement | |  |
| 8. A 30-year-old patient reports waking up one week ago with pain in the left TMJ area and being able to open only ~2 fingers. At your clinical exam, you confirm range of mouth opening of 20 mm with hard endfeel, accompanied by pain on the left TMJ. No TMJ noises are present, but the patient reports a clicking sound with opening in the past. What is the most appropriate diagnosis? | - Disc displacement with reduction of the right TMJ - Disc displacement without reduction of the right TMJ - Disc displacement with reduction of the left TMJ - Disc displacement without reduction of the left TMJ | |  |
| 9. A 25-year-old patient reports that sometimes she gets stuck with her mouth fully open for a few seconds with big yawning. However, she has always been able to close her mouth. What is the most appropriate diagnosis? | - Disc displacement with reduction with intermittent locking - Luxation - Subluxation - Disc displacement without reduction with intermittent locking | |  |
| 10. A “crepitus” on clinical exam with mouth opening and osteoarthritic changes of the condylar head on the CBCT are signs of: | - Disc displacement with reduction - Luxation - Degenerative joint disease of the TMJ - Disc displacement without reduction | |  |
| **Confidence level in TMD-related skills** | | | |
| 1. Screen a dental patient for the presence of TMD | Rate your level of confidence with the following TMD-related skills from 0 to 10, with 10 being the most confident: | | Post-intervention |
|  | Before the activity | After the activity |  |
| 2. Differentiate between TMD pain and dental pain |  |  |  |
| 3. Establish a TMD and orofacial pain diagnosis |  |  |  |
| 4. Explain the diagnosis of TMD and orofacial pain to a patient |  |  |  |
| 5. Identify and explain to the patient the cause of TMD |  |  |  |
| 6. Differentiate between “when to treat” and “when to refer” a TMD cause |  |  |  |
| 7. Educate patients on TMD self-care |  |  |  |
| 8. Insert and adjust a splint for a patient with TMD |  |  |  |
| 9. Perform trigger point injections to confirm your diagnosis |  |  |  |
| 11. Refer and communicate with other healthcare providers about TMD |  |  |  |

TMD: temporomandibular disorders; TMJ: temporomandibular joint.
