# Supplementary material for: Integrating virtual patient-based learning into temporomandibular disorder education improves educational and behavioral outcomes
Source: BMC Med Educ. 2026 May 9;26:1063. doi: 10.1186/s12909-026-09388-0 (PMC13330305; doi:10.1186/s12909-026-09388-0)
Supplement: Supplementary file 2 — Supplementary Material 2. [file 12909_2026_9388_MOESM2_ESM.docx]

**Supplemental File 2**. Sensitivity analysis in baseline study outcomes between participants who completed all study-related tasks and those who completed only the pre-intervention assessment.

|  | **Participants with complete data (n = 77)** | **Participants without complete data (n = 10)** | ***p* value** |
| --- | --- | --- | --- |
| Knowledge performance (mean, SD) | 4.0 ± 1.9 | 4.6 ± 2.1 | .420 |
| Behavioral intervention of TMD screening (N, %)  Very/somewhat likely  Neutral  Mostly/surely unlikely  Unsure  Missing | 55 (71.4%)  16 (20.8%)  5 (6.5%)  1 (1.3%)  0 (0.0%) | 6 (60.0%)  2 (20.0%)  0 (0.0%)  0  2 (20.0%) | .946 |
| Behavioral intervention of TMD management (N, %)  Very/somewhat likely  Neutral  Mostly/surely unlikely  Unsure  Missing | 47 (61.0%)  20 (26.0%)  5 (6.50%)  5 (6.5%)  0 (0.0%) | 5 (50.0%)  3 (30.0%)  0 (0.0%)  0 (0.0%)  2 (20.0%) | .921 |

TMD: temporomandibular disorders.
